# NGS Analysis for Molecular Diagnosis of Retinitis Pigmentosa (RP): Detection of a Novel Variant in *PRPH2* Gene

**DOI:** 10.3390/genes10100792

**Published:** 2019-10-12

**Authors:** Claudia Strafella, Valerio Caputo, Giulia Pagliaroli, Nicola Iozzo, Giulia Campoli, Stefania Carboni, Cristina Peconi, Rosaria Maria Galota, Stefania Zampatti, Giulietta Minozzi, Giuseppe Novelli, Emiliano Giardina, Raffaella Cascella

**Affiliations:** 1 Molecular Genetics Laboratory UILDM, Santa Lucia Foundation, 00179 Rome, Italy; claudia.strafella@gmail.com (C.S.);; 2 Department of Biomedicine and Prevention, Tor Vergata University, 00133 Rome, Italy; 3 Organi di Senso Department, University “la Sapienza”, 00161 Rome, Italy; 4 Department of Veterinary Medicine, University of Milan, 20122 Milan, Italy; 5 Neuromed IRCSS, 86077 Pozzilli, Italy; 6 Department of Biomedical Sciences, Catholic University Our Lady of Good Counsel, 1000 Tirana, Albania

**Keywords:** Retinitis Pigmentosa, NGS panel, *PRPH2*, D2 loop domain, genotype-phenotype correlation

## Abstract

This work describes the application of NGS for molecular diagnosis of RP in a family with a history of severe hypovision. In particular, the proband received a clinical diagnosis of RP on the basis of medical, instrumental examinations and his family history. The proband was subjected to NGS, utilizing a customized panel including 24 genes associated with RP and other retinal dystrophies. The NGS analysis revealed a novel missense variant (c.668T > A, I223N) in *PRPH2* gene, which was investigated by segregation and bioinformatic analysis. The variant is located in the D2 loop domain of PRPH2, which is critical for protein activity. Bioinformatic analysis described the c.668T > A as a likely pathogenic variant. Moreover, a 3D model prediction was performed to better characterize the impact of the variant on the protein, reporting a disruption of the α-helical structures. As a result, the variant protein showed a substantially different conformation with respect to the wild-type PRPH2. The identified variant may therefore affect the oligomerization ability of the D2 loop and, ultimately, hamper PRPH2 proper functioning and localization. In conclusion, *PRPH2*_c.668T > A provided a molecular explanation of RP symptomatology, highlighting the clinical utility of NGS panels to facilitate genotype–phenotype correlations.

## 1. Introduction

Retinitis Pigmentosa (RP, OMIM #268000) includes a group of inherited dystrophies involving the posterior segment of the eye [1]. RP is the most common retinal dystrophy affecting approximately 1:4000 subjects, although prevalence rate depends on geographical localization [2]. RP is one of the leading causes of hypovision and results from degeneration of the retina. Typically, the damage starts in the midperipheral part and progressively extends towards the central portion of retina (macula and fovea). In fact, the damage is caused by an initial loss of rod photoreceptors, followed by the death of cone photoreceptors [3] in the later stages of disease. Phenotypic manifestations of RP include night blindness, impairment of peripheral vision (dysfunction of rod photoreceptors), development of tunnel vision, progressive decrease of the central visual field (cone dysfunction), and dyschromatopsia [2]. From a clinical point of view, the most significant hallmarks of disease are ocular fundus showing dark pigmentary clumps, light-colored retinal vessels, cystoid macular edema, and waxy optic disc pallor [4]. RP can be distinguished in two clinical forms: non-syndromic RP and syndromic RP, which is usually related to other extra-ocular, systemic symptoms. Both types of disorder can be caused by rare mutations in several genes which are inherited according to Mendelian patterns (autosomal dominant, autosomal recessive, X-linked, mitochondrial). To date, more than 80 disease-causing genes have been implicated in RP, which are mostly involved in the alteration of the structure and function of photoreceptors and retinal pigment epithelium. However, incomplete penetrance and variable expressivity generate phenotype and genetic heterogeneity among RP patients [2,5,6]. Generally, RP is diagnosed by clinical (evaluation of visual acuity) and instrumental (electroretinography, visual field testing, optical coherence tomography) analysis. The presence of so many causative genes complicates the selection of reliable and diagnostic genetic assays, although the introduction of Next Generation Sequencing (NGS) represented a critical point for the improvement of RP molecular diagnosis [7]. In particular, NGS gene panels proved to be highly useful to analyze a set of genes associated with a specific disease or a group of related disorders, which are characterized by genetic and phenotypic heterogeneity [8,9]. This is the case of RP, for which the availability of dedicated NGS panels represents one of the best systems to facilitate differential diagnosis, identify new causative mutations and clarify genotype–phenotype correlations. In this report, we describe the application of NGS panel for molecular diagnosis of RP in a family with a clinical history of severe hypovision.

## 2. Materials and Methods 

### 2.1. Clinical Details

**Figure 1:**
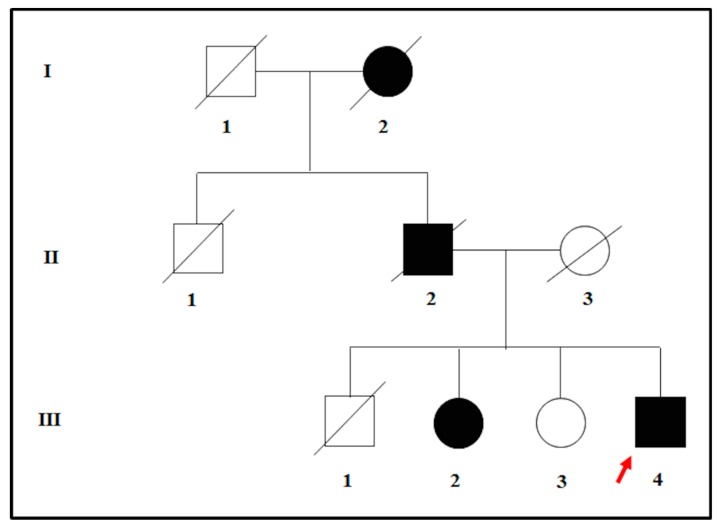
Pedigree tree illustrating the possible autosomal-dominant transmission of disease within the family of the proband. The red arrow indicates the proband.

The proband was affected by a severe hypovision which occurred in adulthood. The clinical assessment was performed at the Sense Organs Department of “Sapienza” University of Rome and included the visual field testing, ocular fundus inspection, Electroretinography (ERG) test, and Optical Coherence Tomography (OCT). At the visual acuity testing, the patient resulted in being completely blind in both eyes. Moreover, the examination of ocular fundus showed the presence of waxy pallor optic disc, attenuated retinal vessels, bone spicule pigment deposits, macular atrophy in the mid-periphery, and posterior pole of eyes. The ERG signal was completely undetectable, and the OCT presented a deeper hyperreflectivity due to the atrophy of neuroepithelium. The patient was therefore diagnosed with non-syndromic RP and was referred to genetic counselling for the molecular confirmation of the clinical diagnosis. During the genetic counseling, the patient revealed that both the deceased father and the paternal grand-mother were blind in both eyes. However, only the father received a clinical diagnosis of RP. Moreover, the proband also had three siblings. One of them was also diagnosed with adult-onset RP whereas the other two were referred to be completely healthy, without any suggestive clinical sign of disease. According to the pedigree (Figure 1) and the family history, an autosomal dominant form of RP (adRP) was hypothesized.

To confirm this hypothesis, the proband and the siblings were subjected to genetic testing to find a molecular basis of their phenotypes. The genetic study was performed according to the Declaration of Helsinki, and all the participants provided signed informed consent. The study was approved by the Ethics Committee of Santa Lucia Foundation (CE/PROG.650 approved on 01/03/2018). 

### 2.2. Laboratory Investigations

Genomic DNA was extracted from 400 µL of peripheral blood using MagPurix Blood DNA Extraction Kit and MagPurix Automatic Extraction System (Resnova) according to the manufacturer’s instructions. The concentration and quality of the extracted DNA was checked by DeNovix Spectrophotometer (Resnova, Rome).

The extracted DNA was sequenced using Ion S5™ System (Ion Torrent™) (ThermoFisher Scientific, Foster City, CA, USA) and Ion Customized Panel High Specificity designed by Ion Ampliseq Designer (ThermoFisher Scientific, Foster City, CA, USA). In this case study, the size of the panel was 16155 Kb and was expected to screen approximately 98.55% of the total panel with a minimum coverage of 20X. The panel included 24 genes, associated with RP and other retinal dystrophies. The selection of the genes was done on the basis of scientific literature, GeneReviews, and considering the frequency of pathogenic variants in the general population. A detailed description of the NGS panel has been summarized in Table 1. 

AmpliSeq libraries were generated using the Ion AmpliSeq™ Library Kit 2.0 (Thermofisher Scientific, Foster City, CA, USA) and processed with Ion Chef™ Instrument (Ion Torrent™, ThermoFisher Scientific, Foster City, CA, USA) for template and enrichment procedures. Samples were subsequently analyzed by Ion S5 System on Ion 520™ Chip (850 flows) (Thermo Fisher Scientific, Foster City, CA, USA). 

Finally, the identified variant was confirmed by direct sequencing performed with BigDye Terminator v3.1, BigDyeX Terminator and ABI3130xl (Applied Biosystem, Warrington, UK) according to the manufacturer’s instructions.

### 2.3. Variant Interpretation

Data analysis and variant annotation were performed by the Ion Reporter Software 5.0 (Life Technologies), Integrative Genomics Viewer (IGV) and TGex software. The interpretation of genetic variants was conducted by Human Gene Mutation Database (HGMD), Leiden Open Variation Database (LOVD), Retinal International, ClinVar, GnomAD, 1000Genomes, and ExAC. 

The functional effect of the detected variants was evaluated by bioinformatic predictive tools such as Mutation Taster, SIFT, PolyPhen 2, Human Splicing Finder (HSF), Varsome, Phyre2, VarSite, and Missense3D. In particular, MutationTaster evaluates the potential pathogenic effect of DNA sequence alterations by predicting the functional consequences of amino acid substitutions, intronic and synonymous alterations, short insertions and/or deletions (indels), and variants spanning intron-exon borders affecting splicing activity [10]. SIFT and PolyPhen2 provide a prediction of the functional effect of amino acid substitutions on proteins [11,12]. HSF predicts the effects of variants on the splicing mechanisms [13]. Varsome is a powerful annotation tool and search engine for human genomic variants, allowing the classification of variants according to ACMG (American College of Medical Genetics) criteria [14]. Phyre2, VarSite and Missense3D are able to analyze the effect of amino acid changes on protein structure, providing a 3D model of the predicted results [15,16,17]. Finally, variants were classified according to the ACMG guidelines, which help provide clinical interpretation of variants, by discriminating among benign, likely benign, uncertain significance, likely pathogenic and pathogenic variants [18].

## 3. Results and Discussion

**Figure 2:**
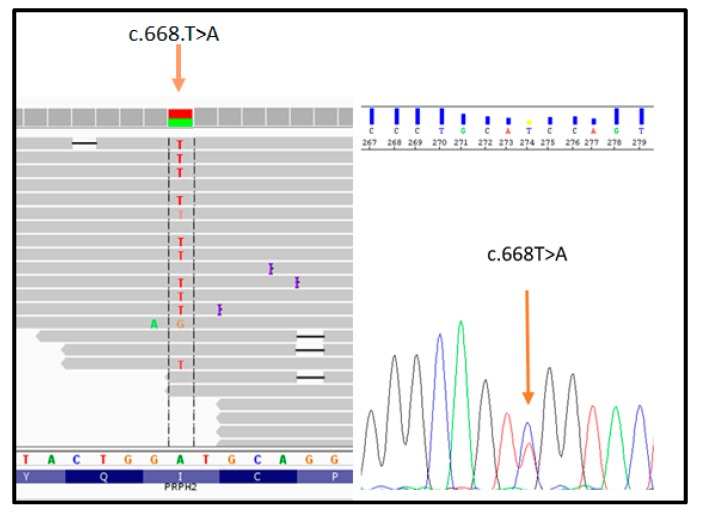
Illustration of the *PRPH2*_c.668T > A variant detected by NGS (on the left) and subsequent confirmation by direct sequencing (on the right).

The proband (patient III: 4 in Figure 1) was analyzed by NGS panel, revealing a novel missense variant (c.668T > A) in *PRPH2* gene at the heterozygous state (Figure 2, left side). The variant was confirmed by direct sequencing (Figure 2, right side).

The c.668T > A results in an amino acid change, namely p.Ile223Asn (I223N). Bioinformatic analysis (Mutation Taster, SIFT, Polyphen2, Varsome, TGex) described c.668T > A as a disease-causing variant. Interrogation of ClinVar, ExAc, LOVD, GnomAD, HGMD, and Retinal International did not report frequency data concerning this variant, suggesting that it has not been described in literature or in any other patient. Given these results, the presence of c.668T > A was tested among the family members of the proband to investigate the familial segregation of the variant. The sequence analysis revealed that the affected sibling (patient III: 2 in Figure 1) carries the same heterozygous variant in *PRPH2*, whereas the healthy sibling (patient III: 3 in Figure 1) was wild-type. According to ACMG guidelines, the c.668T > A can be classified as a likely pathogenic variant, considering that:

- it is not described in the main databases (GnomAD, ExAc and 1000 Genomes) reporting variants frequency in the general population;
- it is located in a gene with a low rate of benign missense variations;
- multiple bioinformatic tools reported c.668T > A as a disease-causing variant;
- it has not been found in more than 100 control tested subjects;
- patient’s phenotype or family history is highly specific for a disease with a single genetic etiology;
- it has been detected in another affected family member;
- *PRPH2* is a known causative gene accounting for ~5–10% of adRP cases [6,19]; and the resulting amino acid change is located within a protein domain harboring other missense variants which are known to be pathogenic for RP [20].

**Figure 3:**
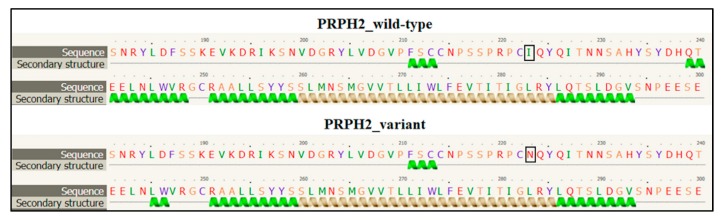
Section of the secondary structure predicted by Phyre2 tool in wild-type and variant PRPH2 sequence. The black square indicates the position of the amino acid change. The figure shows the disruption of the α-helical structure between the residues 239 and 250 in the variant PRPH2 with respect to the wild-type. The other residues reporting the altered secondary structure are not shown.

**Figure 4:**
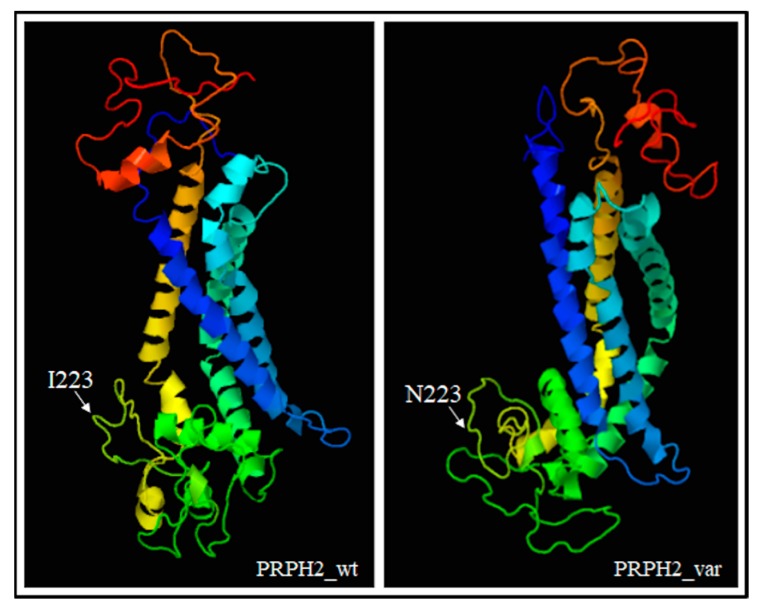
3D models showing the different conformation of variant PRPH2 with respect to the wild-type protein. The position of the residue 223 has also been indicated. The prediction was based on ctxc5a (PDB header: cell invasion; PDB Title: crystal structure of human tetraspanin cd81). Wt: wild-type, var: variant.

*PRPH2* encodes a transmembrane glycoprotein called Peripherin-2/Retinal Degeneration Slow (PRPH2/RDS, hereafter referred to as PRPH2), which is critical for the morphogenesis, maintenance and stabilization of the disc rims of the outer segments in rod and cone photoreceptors. PRPH2 is able to interact with itself and its homologue Rod Outer Segment Membrane protein 1 (ROM-1). PRPH2 and ROM-1 can interact together, forming homo- and hetero-tetramers which are further connected by disulphide bounds to constitute high-order oligomers and allow disc rim formation [21]. One of the most important domains of PRPH2 is the large D2 loop domain that extends for 142 amino acids (from the 125^th^ to the 163^rd^ residue of protein) and is normally located within the intradiscal part of the rim [20]. Considering the positioning of the I223N within PRPH2, the variant has been furtherly investigated with Phyre2, Varsite and 3D missense bioinformatic tools that are able to analyze the effect of amino acid changes on protein structure, providing a 3D model of the predicted results. The prediction analysis showed that the amino acid substitution of an Asparagine residue (N, Asn) with an Isoleucine (I; Ile) at the 223^rd^ residue may be highly negative in terms of conserved amino acid properties and, thus, modify the secondary and tertiary structures of PRPH2. Concerning this hypothesis, it is important to remark that Ile is a non-polar hydrophobic amino acid whereas Asn is a polar and hydrophilic residue, which may thereby alter the conformation and the localization of the protein. The Phyre2 analysis predicted a disruption of the α-helical structures (in the residues 10–17, 86–95, 150–167, 239–250) within the variant protein (Figure 3), leading to a substantial different conformation with respect to the wild-type (Figure 4). 

The altered conformation resulting from the amino acid substitution may therefore affect the oligomerization ability of the D2 loop and, consequently, hamper the proper functioning and cellular localization of PRPH2. Altogether, these findings supported the pathogenic effect of c.668T > A in the proband and the affected sibling, although functional assays are necessary to confirm the real impact of this variant on RP etiopathogenesis.

Supporting our results, different mutations have already been described within the D2 domain. Similarly to our variant, most of them are missense, are localized within a specific D2 loop region spanning from Lys193 to Glu226, and have been described as pathogenic for late-onset adRP [20,21,22]. However, *PRPH2* variants have also been involved in a wide range of autosomal dominant retinal disorders, including RP, cone-rod dystrophy, adult vitelliform macular dystrophy, cone dystrophy, and pattern dystrophy [20,21,22]. Such a high genetic heterogeneity further complicates the genotype–phenotype correlations. In the present study, the genetic analysis was consistent with the clinical diagnosis of RP, which has been probably transmitted by an autosomal-dominant pattern within the family. This work described a novel variant in *PRPH2* as a possible pathogenic mutation for adRP, providing additional knowledge about the involvement of the D2 loop domain of PRPH2 in the etiopathogenesis of retinal disorders. Moreover, the present study illustrates the clinical utility of NGS panels to facilitate the genotype–phenotype correlations in retinopathies characterized by high genetic heterogeneity and variable expressivity. The availability of analytical software for discriminating gene variants on the basis of specific phenotype/disorders can improve the accuracy of the interpretation and reduce the time required for providing the final response. From this perspective, individual genetic profiles can be extremely helpful in combination with clinical and instrumental data to define a comprehensive picture of the patient and calculate the recurrence risk of disease within the family and, subsequently, in the offspring of affected members [23,24].

## Figures and Tables

**Table 1:** Customized NGS panel utilized for molecular diagnosis of RP and other retinal dystrophies.

Gene	Locus	OMIM	Size of the Target (bp)	Exons	Transcript ID	Coverage (%)
*RHO*	3q22.1	180380	1147	5	ENST00000296271.3	100
*PRPF31*	19q13.42	606419	1760	14	ENST00000321030.8	100
*PRPH2*	6p21.1	179605	1101	3	ENST00000230381.6	100
*RP1*	8q11.2-q12.1	603937	6531	4	ENST00000220676.1	100
*IMPDH1*	7q32.1	146690	2154	17	ENST00000338791.10	88.21
*PRPF8*	17p13.3	607300	7848	43	ENST00000304992.10	100
*KLHL7*	7p15.3	611119	2060	11	ENST00000339077.9	100
*NR2E3*	15q23	604485	1398	8	ENST00000617575.4	100
*CRX*	19q13.33	602225	960	4	ENST00000221996.11	91.77
*PRPF3*	1q21.2	607301	2352	16	ENST00000324862.6	100
*TOPORS*	9p21.1	609507	3198	3	ENST00000360538.6	100
*USH2A*	1q41	608400	17043	72	ENST00000307340.7	100
*ABCA4*	1p22.1	601691	7822	50	ENST00000370225.3	100
*PDE6A*	5q32	180071	3023	22	ENST00000255266.9	100
*PDE6B*	4p16.3	180072	3005	22	ENST00000496514.5	98.2
*RPE65*	1p31.3	180069	1882	14	ENST00000262340.5	100
*CNGA1*	4p12	123825	2460	10	ENST00000402813.7	100
*BEST1*	11q12.3	607854	2214	9	ENST00000449131.6	100
*SEMA4A*	1q22	607292	2566	15	ENST00000368285.7	99.96
*EYS*	6q12	612424	10368	43	ENST00000503581.5	100
*CRB1*	1q31.3	604210	4616	12	ENST00000367400.7	100
*CERKL*	2q31.3	608381	1957	13	ENST00000410087.7	90.5
*RPGR*	Xp11.4	312610	4382	15	ENST00000378505.7	82.25
*RP2*	Xp11.3	300757	1153	5	ENST00000218340.3	100

## References

[1] Cascella R., Strafella C., Germani C., Novelli G., Ricci F., Zampatti S., Giardina E. (2015). The Genetics and the Genomics of Primary Congenital Glaucoma. Biomed. Res. Int..

[2] Verbakel S.K., van Huet R.A.C., Boon C.J.F., den Hollander A.I., Collin R.W.J., Klaver C.C.W., Hoyng C.B., Roepman R., Klevering B.J. (2018). Non-syndromic retinitis pigmentosa. Prog. Retin. Eye Res..

[3] Cascella R., Strafella C., Longo G., Ragazzo M., Manzo L., De Felici C., Errichiello V., Caputo V., Viola F., Eandi C.M. (2017). Uncovering genetic and non-genetic biomarkers specific for exudative age-related macular degeneration: significant association of twelve variants. Oncotarget.

[4] Ali M.U., Rahman M.S.U., Cao J. (2017). Genetic characterization and disease mechanism of retinitis pigmentosa; current scenario. 3Biotech.

[5] Fiorentino A., Yu J., Arno G., Pontikos N., Halford S., Broadgate S., Michaelides M., Carss K.J., Raymond F.L., Cheetham M.E. (2018). Novel homozygous splicing mutations in ARL2BP cause autosomal recessive retinitis pigmentosa. Mol. Vis..

[6] Dias M.F., Joo K., Kemp J.A., Fialho S.L., da Silva Cunha A., Woo S.J., Kwon Y.J. (2018). Molecular genetics and emerging therapies for retinitis pigmentosa: Basic research and clinical perspectives. Prog. Retin. Eye Res..

[7] Birtel J., Gliem M., Mangold E., Müller P.L., Holz F.G., Neuhaus C., Lenzner S., Zahnleiter D., Betz C., Eisenberger T. (2018). Next-generation sequencing identifies unexpected genotype-phenotype correlations in patients with retinitis pigmentosa. PLoS ONE.

[8] Adams D.R., Eng C.M. (2019). Next-generation sequencing to diagnose suspected genetic disorders. N. Engl. J. Med..

[9] Strafella C., Campoli G., Galota R.M., Caputo V., Pagliaroli G., Carboni S., Zampatti S., Peconi C., Mela J., Sancricca C. (2019). Limb-Girdle Muscular Dystrophies (LGMDs): The Clinical Application of NGS Analysis, a Family Case Report. Front Neurol..

[10] Schwarz J.M., Cooper D.N., Schuelke M., Seelow D. (2014). MutationTaster2: Mutation prediction for the deep-sequencing age. Nat. Methods.

[11] Sim N.L., Kumar P., Hu J., Henikoff S., Schneider G., Ng P.C. (2012). SIFT web server: Predicting effects of amino acid substitutions on proteins. Nucleic Acids Res..

[12] Adzhubei I., Jordan D.M., Sunyaev S.R. (2013). Predicting functional effect of human missense mutations using PolyPhen-2. Curr. Protoc. Hum. Genet..

[13] Desmet F.O., Hamroun D., Lalande M., Collod-Béroud G., Claustres M., Béroud C. (2009). Human Splicing Finder: an online bioinformatics tool to predict splicing signals. Nucleic Acids Res..

[14] Kopanos C., Tsiolkas V., Kouris A., Chapple C.E., Albarca Aguilera M., Meyer R., Massouras A. (2018). VarSome: The Human Genomic Variant Search Engine. Bioinformatics.

[15] Kelley L.A., Mezulis S., Yates C.M., Wass M.N., Sternberg M.J. (2015). The Phyre2 web portal for protein modeling, prediction and analysis. Nat. Protoc..

[16] PDBsum: Pictorial database of 3D structures in the Protein Data Bank. https://www.ebi.ac.uk/thornton-srv/databases/cgi-bin/DisaStr/GetPage.pl?uniprot_acc=n/a&template=home.html.

[17] Ittisoponpisan S., Islam S.A., Khanna T., Alhuzimi E., David A., Sternberg M.J.E. (2019). Can Predicted Protein 3D Structures Provide Reliable Insights into whether Missense Variants Are Disease Associated?. J. Mol. Biol..

[18] Richards S., Aziz N., Bale S., Bick D., Das S., Gastier-Foster J., Grody W.W., Hegde M., Lyon E., Spector E. (2015). ACMG Laboratory Quality Assurance Committee. Standards and guidelines for the interpretation of sequence variants: A joint consensus recommendation of the American College of Medical Genetics and Genomics and the Association for Molecular Pathology. Genet. Med..

[19] Manes G., Guillaumie T., Vos W.L., Devos A., Audo I., Zeitz C., Marquette V., Zanlonghi X., Defoort-Dhellemmes S., Puech B. (2015). High prevalence of PRPH2 in autosomal dominant retinitis pigmentosa in france and characterization of biochemical and clinical features. Am. J. Ophthalmol..

[20] Boon C.J., den Hollander A.I., Hoyng C.B., Cremers F.P., Klevering B.J., Keunen J.E. (2008). The spectrum of retinal dystrophies caused by mutations in the peripherin/RDS gene. Prog. Retin. Eye Res..

[21] Chakraborty D., Rodgers K.K., Conley S.M., Naash M.I. (2013). Structural characterization of the second intra-discal loop of the photoreceptor tetraspanin RDS. FEBS J..

[22] Stuck M.W., Conley S.M., Naash M.I. (2016). PRPH2/RDS and ROM-1: Historical context, current views and future considerations. Prog. Retin. Eye Res..

[23] Cascella R., Strafella C., Longo G., Manzo L., Ragazzo M., De Felici C., Gambardella S., Marsella M.L.T., Novelli G., Borgiani P. (2017). Assessing individual risk for AMD with genetic counseling, family history, and genetic testing. Eye.

[24] Strafella C., Caputo V., Galota M.R., Zampatti S., Marella G., Mauriello S., Cascella R., Giardina E. (2018). Application of precision medicine in neurodegenerative diseases. Front Neurol..

